# Functional and phylogenetic diversity of an agricultural matrix avifauna: The role of habitat heterogeneity in Afrotropical farmland

**DOI:** 10.1002/ece3.9024

**Published:** 2022-07-06

**Authors:** Marie Laure Rurangwa, Protais Niyigaba, Joseph A. Tobias, Robert J. Whittaker

**Affiliations:** ^1^ Wildlife Trust Rwanda Kigali Rwanda; ^2^ Wildlife Conservation Society – Rwanda Kigali Rwanda; ^3^ Faculty of Natural Sciences, Department of Life Sciences Imperial College London Berks UK; ^4^ School of Geography and the Environment University of Oxford Oxford UK; ^5^ Center for Macroecology, Evolution and Climate, GLOBE Institute University of Copenhagen Copenhagen Denmark

**Keywords:** agrobiodiversity, agroforestry, birds, farmland heterogeneity, functional diversity, phylogenetic diversity, Rwanda

## Abstract

Varied strategies to alleviate the loss of farmland biodiversity have been tested, yet there is still insufficient evidence supporting their effectiveness, especially when considering phylogenetic and functional diversity alongside traditional taxonomic diversity metrics. This conservation challenge is accentuated in the Afrotropics by the rapid agricultural expansion and intensification for the production of cash crops and by a comparative lack of research. In this study, we assessed how farming practices influence avian phylogenetic and functional diversity. We conducted point‐count surveys to assess avian diversity in monocultures of tea and mixed crop farming systems surrounding the Nyungwe rainforest in south‐west Rwanda, allowing us to investigate the drivers of avian diversity at farm level. Species composition was found to be moderately different between farm types, with mixed crop farms supporting higher phylogenetic diversity than tea plantations. There were no significant seasonal differences in species composition, functional or phylogenetic diversity. Overall, functional diversity did not differ between farm types, but the dispersion of trophic‐related traits was significantly higher in mixed crop farms. Both functional and phylogenetic diversity were influenced by floristic diversity, vegetation height, tree number, and elevation to varying degrees. Our results also (i) highlight the role of farmland heterogeneity (e.g., crop species composition, height, and tree cover extent) in encouraging avian functional and phylogenetic diversity in the Afrotropics and (ii) indicate that the generally negative biodiversity impacts of monoculture agriculture can be partially alleviated by extensive agroforestry with an emphasis on indigenous tree species.

## Graphical abstract

We studied avian diversity in a tropical agricultural matrix and found that mixed crop farms supported higher phylogenetic diversity, and greater dispersion of trophic‐related traits than tea plantations owing to the influence of crop species composition, height, and Agroforestry practices.

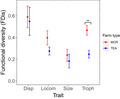

## 1 INTRODUCTION

Extensive agriculture, practiced at both subsistence and industrial levels, is among the leading drivers of biodiversity loss, particularly in the tropics and subtropics (Diaz et al., 2019; Malhi et al., 2014). The environmental impacts of food production are exacerbated by global trade interconnectivity and high consumerism in developed countries. For example, Chaudhary et al. (2016) showed that 95% of “biodiversity damage” attributable to Swiss food consumption in 2011 happened abroad, mostly in the tropics. These impacts were evaluated at a global level from estimates of species loss per unit area due to a given land‐use, and were found to be up to 300 times greater than the damage resulting from domestic agriculture. A key factor is that tropical cash crops, such as coffee, palm oil, rubber, tea, and soybean, may be cultivated on a much smaller area than domestic cereals such as rice, maize, and wheat, yet nonetheless inflict disproportionately high damage on biodiversity because they tend to be grown in areas supporting substantial numbers of endemic and threatened species (Chaudhary et al., 2016).

The production of cash crops contributes to biodiversity loss largely through forest clearance, as well as the reduction of crop diversity since they are often grown as extensive monocultures (Jiang et al., 2019; Lees et al., 2015; Perfecto et al., 2019). For example, Xu et al. (2018) reported that from 2010 to 2015, 9335 ha of tea plantations were established in Menghai county, China, representing a 33.6% increase within five years. One third of the land converted was obtained by deforestation. A similar trend was observed in the Eastern Himalaya piedmont between 1874 and 2010, where a fall of 69.5% in forest cover was accompanied by an increase of 30.7% in tea plantations, independent of population growth (Prokop, 2018). Global tea production is rising, even in small countries, such as Rwanda, which registered the highest global annual growth rate of 26.8% between 2007 and 2016 (IGT, 2018). With the onset of the COVID‐19 pandemic, tea surpassed tourism as the leading earner of export revenues in Rwanda. In a single year (2019–2020), increased tea production in Rwanda generated export revenues of US $83,552,108, marking a growth of 12% compared to the previous fiscal year (NAEB, 2020). The growing “ecological footprint” of tea production is thus an important factor contributing to habitat change in the tropics, where all the top‐producing countries are located.

The need to conserve farmland biodiversity has led to the development of conservation programs underpinned by the concept that heterogeneous landscapes—which comprise diverse habitats (compositional heterogeneity), varying in their spatial patterning (configurational heterogeneity)—will provide greater and complementary resources, facilitate dispersal processes and enable the coexistence of species of diverse functional strategies (Batáry et al., 2015; Fahrig et al., 2011). In Europe, agri‐environment schemes of the European Union's Common Agricultural Policy enable farmers within member states to access financial support to maintain or adopt measures that curb biodiversity loss (particularly of bird species) and which sustain ecosystem functions (Chamberlain, 2018; Kleijn & Sutherland, 2003). In the tropics, the adoption of agroforestry policies is gaining traction among governments and private stakeholders, with the aim of attaining biodiversity goals, and responding to socio‐economic needs, such as the need for fuelwood (Mukuralinda et al., 2016; Şekercioğlu, 2012).

Although a wide range of mechanisms to curb biodiversity loss have been trialed in different countries, there is still little evidence attesting to their effectiveness. Studies on agri‐environment schemes have produced contrasting results (Batáry et al., 2015; Hiron et al., 2015; Lee & Goodale, 2018). Although the role of agroforestry systems in maintaining avian diversity in tea plantations has been demonstrated (Chetana & Ganesh, 2012; Sidhu et al., 2010), the basis of assessment has largely been the taxonomic diversity of the bird communities, a relatively simplistic metric that provides limited information about how ecosystems function and respond to environmental disturbances compared to other components of avian diversity. For example, components of avian diversity, such as functional and phylogenetic diversity, are expected to more closely reflect ecosystem function and resilience (Cadotte et al., 2011; Hordley et al., 2021; Sheard et al., 2020). Previous studies focusing on the impacts of tropical agriculture on functional and phylogenetic diversity have mainly focused on the impact of pasture, palm oil, and coffee (e.g., Cannon et al., 2019; Chapman et al., 2018). As a result, the impacts of tea agricultural practices remain poorly understood, particularly in the Afrotropics.

This study was conducted in Rwanda, a country whose vision to attain a “green” economy is challenged by a high population density (477 people per km^2^), with more than 80% of the population carrying out subsistence farming (NISR, 2018). We focused on birds due to their diverse ecological niches, the range of farmland services they contribute to (e.g., pollination, pest control, and seed dispersal), their value as ecosystem health indicators (Catterall et al., 2012; Şekercioğlu, 2012; Sinu, 2011), and the availability of data on their diversity in the study area. Due to the marked differences in the vegetation composition characterizing tea and mixed‐crop farms, we hypothesized that: (i) distinct avian communities will be associated with the two farming systems; (ii) the greater structural and compositional complexity of mixed crops will result in higher functional and phylogenetic diversity values for mixed crop farms compared to tea farms; and (iii) functional and phylogenetic diversity will be driven by different habitat attributes, with crop heterogeneity and tree coverage being key factors.

## 2 MATERIALS AND METHODS

### 2.1 Study area

**FIGURE 1:**
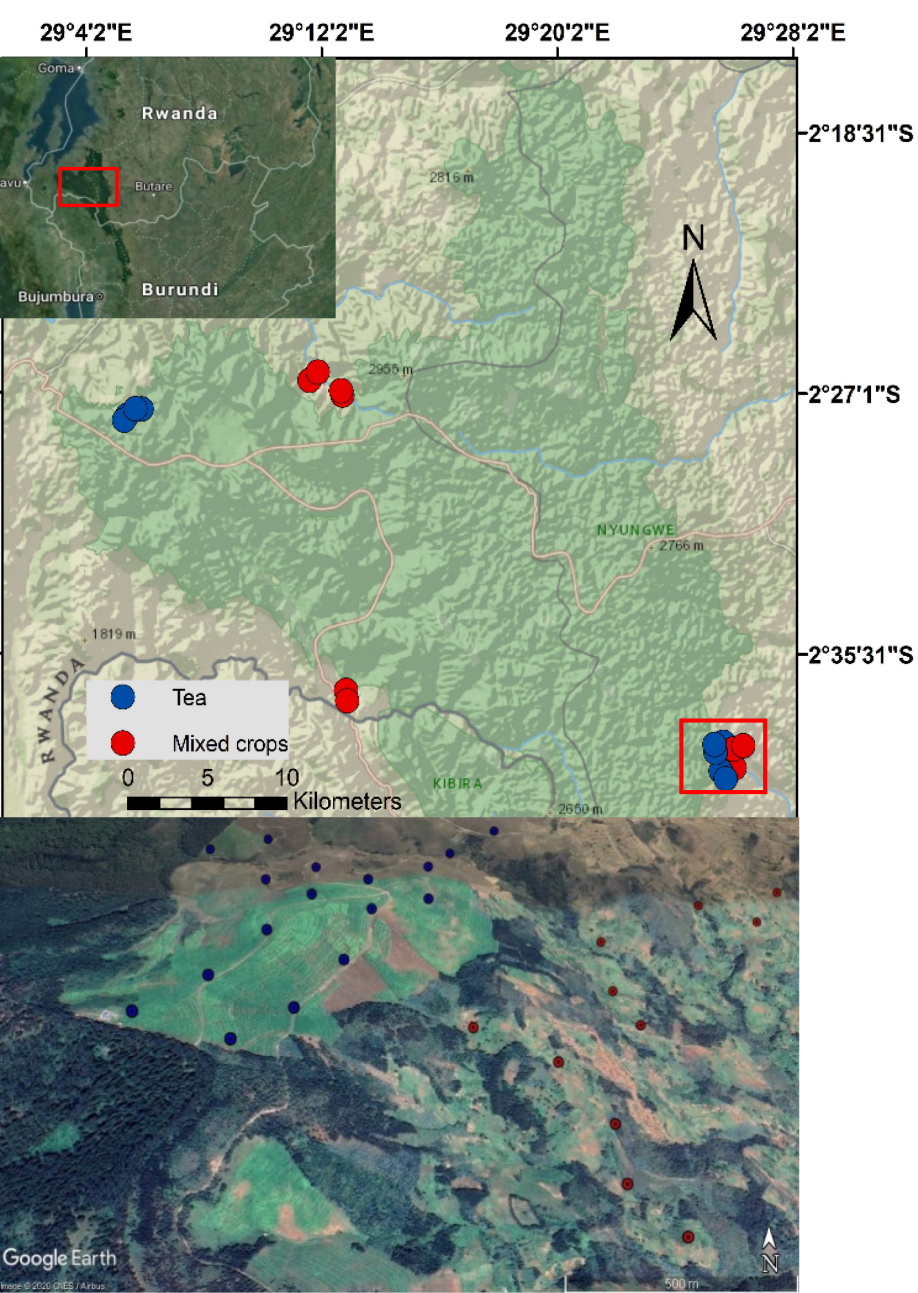
Geographic location of 100 point counts (20 samples) in tea and mixed crops farms around the Nyungwe National Park, Rwanda. The Nyungwe forest adjoins Kibira National Park, Burundi. Each point was sampled twice, once in the wet and once in the dry season between November 2017 and August 2018. Basemap and satellite image sources: National Geographic, and Google Earth

The study was conducted in agricultural areas surrounding Nyungwe National Park, hereafter Nyungwe NP, a tropical montane rainforest located in south‐west Rwanda (Figure 1). Mean annual rainfall is 1500–2500 mm, and annual temperature varies between an average minimum of 10.9°C and an average maximum of 19.60°C (Sun et al., 1996). Two major farming types could be distinguished from the field. Monocultures of tea (*Camellia sinensis*) grown by tea companies and individual farmers, and farms containing mixed crops, mainly maize, beans (*Phaseolus vulgaris*), sweet potatoes, and cassava. Outside the forest, farming was the predominant land‐use, followed by settlements, which increased in density with distance from the forest. Tea estates were established after government‐supported forest clearance in the 1960s. In addition to plantations owned by the government and private factories, farmers have been encouraged to grow tea on their own lands to increase the total production. On lands owned by private farmers, tea was often grown close to other subsistence crops. Due to the reinforced protection of the forest since 2004, and scarcity of land, the average household farm size around Nyungwe NP is around 1 ha (Masozera & Alavalapati, 2004). To encourage improved economic viability of land use, the Rwandan government introduced the land use consolidation act in 2007, which urged farmers with adjacent lands to grow a single priority crop in a given season. Although, mixed‐crop farms mostly contained at least two crops, adjoining farms in the study area tended to grow the same crops, making it difficult to distinguish individual farms.

### 2.2 Bird surveys

Bird communities were sampled using standard methods of point‐count sampling commonly applied in tropical systems (Bibby et al., 2000; Leach et al., 2016; Vollstädt et al., 2017). Although surveying birds in tropical landscapes is challenging (Robinson et al., 2018), we focused on relatively open farmland habitats, where most bird species are fairly detectable. To choose sampling sites, we organized consultative meetings with key people engaged in the management and monitoring of Nyungwe NP and surrounding areas. The sites were selected based on safety and accessibility. For further information on the study design, see Rurangwa et al. (2021).

We established 100 point‐count stations (50 in tea plantations; 50 in mixed crops farms). The starting point was randomly selected, and each point was separated by an interval of 200 m from the previous point to increase statistical independence by minimizing double counting (Ralph et al., 1995). At each point, birds seen or heard within a 20‐m radius were recorded for 10 min. Observations were replicated in two seasons, producing a total of 200 point counts for the whole study. We sampled in both dry and wet seasons, from November 2017 to February 2018, and then from June to August 2018. Surveys began at 6 a.m. and ended by 10 a.m., with slightly later start‐times for sites located far from the main road. No surveys were undertaken during rain. Survey data were recorded by one observer with more than 20 years of local experience.

### 2.3 Avian functional traits collection

Functional traits of birds in the study area relating to morphology and habitat use were extracted from a published dataset (Tobias et al., 2022). Morphological measurements were taken in the field and from specimens housed at museums and collections worldwide, mainly the Natural History Museum at Tring (for the sampling protocol, see Tobias et al., 2022). Beak length (measured along culmen and from nares), as well as width and depth measured at the nares, were included as an index of the trophic niche (Pigot et al., 2020; Schoener, 1965); wing length, and Kipp's distance (the linear distance between the tips of the longest primary and the first secondary measured on a folded wing), as well as tarsus length, were included to represent dispersal capabilities and locomotion related to foraging behavior (Pigot et al., 2020; Sheard et al., 2020). Biometric measures were taken where possible from two adult male and two adult female specimens per species. Foraging strata were directly recorded from the field and supplementary information was obtained from Vande weghe and Vande weghe (2011). The foraging strata were categorized as lower (3 m and below), middle (4–7 m), and upper (>7 m). Dietary data for each species were retrieved from Wilman et al. (2014), who compiled proportions of the diet of each species in terms of major food categories, including: fruit, invertebrates, nectar, omnivores, plant matter, vertebrates, and seeds.

### 2.4 Farm assessment

We established sampling points in tea and mixed crop farms. Tea farms were often characterized by monoculture, while in mixed crop farms at least two crops were grown, and at times plots were separated by houses.

For each point we sampled, we assessed the floristic diversity to represent the vegetation composition, and crop height and the number of trees to represent the vegetation structure. We included elevation since it has been found to influence different components of the avian diversity, albeit to varying degrees (Ding et al., 2021; Hanz et al., 2019; Rurangwa et al., 2021). Differences in elevation also underpin patterns of variation in farming practices, such as the classification of agro‐ecological zones in Rwanda. For instance, tea plantations are established on acidic soils in mountainous areas (1500–2500 m). We also recorded temperature, relative humidity, and soil moisture due to the relationship between microclimatic and edaphic factors and the conditions for habitat use by birds, such as the abundance of prey, and suitable nesting sites (Cifuentes‐Croquevielle et al., 2020; Sutherland & Green, 2004; Visco et al., 2015). We recorded the distance between plots and the Nyungwe forest, since proximity to forests has been found to permit the persistence of forest‐affiliated avian communities in agricultural landscapes (Cannon et al., 2019; Socolar et al., 2019).

We recorded the number and species identity of all plants within the radius of each point count, and the number of trees with stem Diameter at Breast Height >5 cm. The crop height (including noncrop species) was measured with a three‐meter folding rule. We measured the temperature and humidity using a portable data logger, and soil moisture using a soil moisture probe. The application of agro‐chemicals was not assessed due to the low‐intensity farming methods practiced and the sporadic documentation of agro‐chemicals when applied (Appendix S1). Measurements at each point were taken once per season by two field botanists with more than 15 years of experience.

### 2.5 Statistical analysis

We performed a Detrended Correspondence Analysis (Hill, 1979) to explore avian community composition within tea and mixed crops farming systems. We further conducted the analysis of similarity (ANOSIM) test using the CAP program (Seaby et al., 2014) to determine if samples within each farming type had a greater similarity than that occurring by chance. Statistical analyses were conducted at a sample level. Where seasonal effects were to be investigated, a sample comprised 5 contiguous points within the same area, sampled the same day in each season, amounting to 40 samples per study. Otherwise, the values of the two seasons were averaged to avoid pseudo‐replication, giving 20 samples.

We used the near tool from ArcGis 10.5.1 (ESRI, 2019) to calculate the nearest geodesic distance between the sample centroid and the Nyungwe forest. The sample centroid was calculated using the Mean Center tool from ArcGIS, which averages the longitude and latitude coordinates of the five points.

To compare functional diversity and phylogenetic diversity between farm type, we used metrics that provided complementary information and incorporated abundances.

Functional dispersion (FDis)—Measures the spread of species traits by quantifying the mean distance of each species to the centroid of all species weighted by abundance (Laliberté & Legendre, 2010). One benefit of FDis as a metric is that it allows retention of samples with fewer than three observations, which can occur at finer scales of sampling.

Phylogenetic diversity (^q^PD[*T*])—The total diversity of branches of a phylogeny based on Hill numbers. q denotes the order of magnitude of abundance (q = 0 excludes abundance, and only species richness is considered, q = 1 considers common species, q = 2 includes only the most abundant species (Chao et al., 2010).

Standardized effect size of Mean Pairwise distance (sesMPD)—The average pairwise phylogenetic distances among individuals in a community, adjusted for species richness. Higher values indicate that the community is composed of species which are evenly distributed across clades, while lower values indicate a community constituted of species that are phylogenetically clustered (Webb et al., 2002).

Standardized effect size of Mean nearest Taxon distance (sesMNTD)—The average phylogenetic distance between an individual and its closest relative. The metric reveals phylogenetic structuring at the tips of the tree. A community that does not contain closely related individuals will have higher sesMNTD values, and the opposite is true for lower sesMNTD values (Webb et al., 2002).

Due to the high correlation of avian morphological traits (caused largely by body mass), and high intraspecific variations, a series of Principal Components Analyses (PCA) were performed, and resultant axes were used as indices of body size, trophic processes, locomotory abilities, and flight and dispersal capabilities, following Trisos et al. (2014). FDis was then computed from a matrix containing the obtained indices, diet, and foraging strata information using the R package FD (Laliberté et al., 2014). Quantitative traits were first rescaled (mean = 0, s.d. = 1) prior to the computation of FDis.

To analyze phylogenetic diversity, we downloaded 1000 trees from a global avian phylogeny (Jetz et al., 2012; www.birdtree.org accessed 20 June 2019) based on the Ericson backbone (Ericson et al., 2006), and calculated PD(*T*) metrics using HillR packages (Li, 2018), and the sesMPD, and sesMNTD, using the picante package (Kembel et al., 2010).

Univariate type III repeated‐measures ANOVA were used to compare diversity metrics across farm types and within seasons and *t*‐tests were used to investigate variations in ses.MNTD, ses.MPD, and differences in subsets of trophic‐related traits across farm types. To analyze the effects of farm attributes on PD and FD, two different multiple linear regression models were performed for ^1^PD(T) and FDis containing floristic diversity, crop height, the number of trees, humidity, soil moisture, elevation, and distance to the Nyungwe forest from the sample centroid as explanatory variables. Floristic diversity was computed as the exponential Shannon‐Wiener index based on raw abundances of all crop and noncrop plant species found in each point station. Temperature and tree height were not included in the models due to their high correlation with humidity and vegetation height, respectively (Pearson's *R* > = 0.7, Table S1).

We further employed a stepwise model selection procedure, based on the Akaike information criterion adjusted for sample size (AIC_c_) using the MuMin package (Anderson & Burnham, 2004; Barton, 2019). The final model was obtained from averaging models within ΔAIC_c_ < 2 of the model with the lowest AIC_c_ value. Spatial autocorrelation was evaluated on both model residuals using Moran's I test and it was found to be nonsignificant for both ^1^PD(*T*) and FDis (*p* = .082 and .44, respectively). All analyses were conducted in R 3.5.3 (R Core Team, 2019).

## 3 RESULTS

### 3.1 Community structure

We recorded 755 individuals belonging to 63 bird species, including three sunbirds endemic to the Albertine Rift region: *Nectarinia purpureiventris, Cinnyris regius,* and *C. stuhlmannii* (Table S2). Fifty species were found in mixed crops, 33 in tea farms: 20 species were found in sites from both farm categories. The most abundant species were *Crithagra citrinelloides* and *Psalidoprocne pristoptera,* which constituted 11.7% and 9.4% of total abundances, respectively, across the two sampling periods. The dominant guild was the insectivores, with 27 species out of 63. Within farm type, the pattern persisted: small insectivores were the most commonly recorded guild in terms of species richness, while granivores dominated in terms of abundances in mixed crop farms (Table 1). The Nyungwe NP provided the source for some forest species that strayed into the more open agricultural habitats, including the endemic birds we have mentioned. The majority of these species were generalists. In total, 11 species were shared between the forest and tea farms, and 31 species between the forest and mixed crop farms.

**TABLE 1:** Raw abundances of feeding and size guilds of birds recorded in farms around Nyungwe NP, Rwanda. Sunbirds are classified as omnivores because they consume both invertebrates and nectar. The dietary classification follows Wilman et al. (2014). Small: <44 g; medium: 45‐200 g; large: > 201 g. numbers given in brackets are the percentage of abundances computed within each farm type

	Invertebrates	FruitNect	Omnivore	Plant/seed	VertFishSc
Mixed crops					
Small	20 (32.7)	4 (8.5)	5 (15.91)	10 (35.8)	
Medium		1 (1.27)	1 (1.63)	2 (0.36)	
Large	1 (0.72)	2 (1.27)	1 (0.36)		3 (1.45)
Tea					
Small	12 (40.1)	2 (1.49)	8 (11.89)	4 (30.2)	
Medium	1 (0.5)		2 (4.46)	2 (1.49)	
Large		1 (6.93)			1 (2.97)

*Note*: FruitNect denotes birds feeding on fruits and/or nectar, while VertFishSc denotes birds feeding or scavenging on vertebrates, including fish.

**FIGURE 2:**
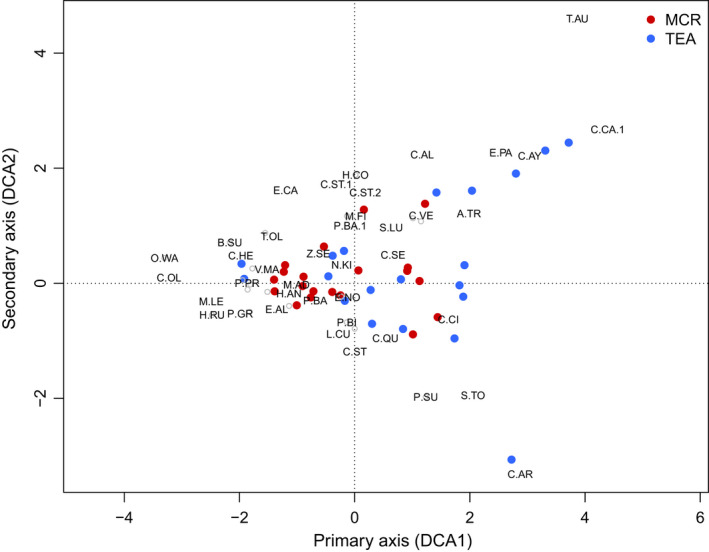
Biplot of Detrended correspondence analysis axes 1 and 2, showing bird species composition in 40 samples (each comprising 5 point counts sampled on the same day in close proximity) within tea and mixed crop farms around Nyungwe National Park, Rwanda in 2017–2018. The data comprise 20 such samples from November 2017 to February 2018, and the same sites resampled between June and August 2018. For readability, in cases of overlap, only the species with a higher occurrence value is displayed. For full species names, the reader is referred to Table S2. The outlying species *Columba arquatrix* (C.AR) was recorded at an unusually high number of 12, while *Tchagra australis* (T.AU) was the only species in its sample

**FIGURE 3:**
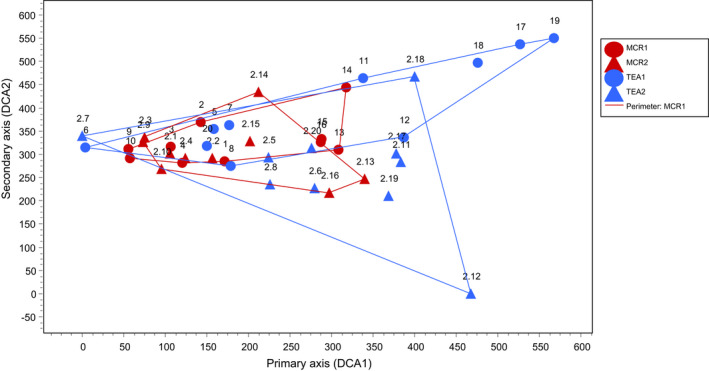
Detrended correspondence analysis plot showing sample site scores (*N* = 40) along axis 1 and 2 based on raw abundances of birds surveyed in tea (blue) and mixed crops (red) around Nyungwe NP during the wet season (1) and dry season (2). MCR denotes mixed crops. The outlying sample 2.12 comprised only 2 species, one of them (*Columba arquatrix*) was recorded in high numbers. Samples 17 and 18 had only two species: *Cisticola ayresi* and *Cossypha caffra*. Sample 18 had only 2 individuals, the lowest abundance value

The turnover of species among samples per season was high, as evidenced by the length of the DCA axes being above four standard deviation units (Jongman et al., 1995), with the first and second axes mostly reflecting elevation (Figure 2; Figure S1). The DCA shows a degree of overlap among the farm types. This was confirmed by the ANOSIM test, which revealed samples within the farm types were moderately distinct (ANOSIM sample statistic *R* = 0.299, Table S3), however, the differences were not statistically significant between seasons (Figure 3, Table S3). Tea samples were more spread out, but the extremes were dominated by samples characterized by few species (in terms of encounter rate), particularly those normally affiliated to gallery forest, scrub, and other habitats adjacent to forest, including montane grasslands, such as *Columba arquatrix*, *Cisticola ayresi,* and *Cossypha caffra*.

### 3.2 Phylogenetic and functional diversity between farm types

**FIGURE 4:**
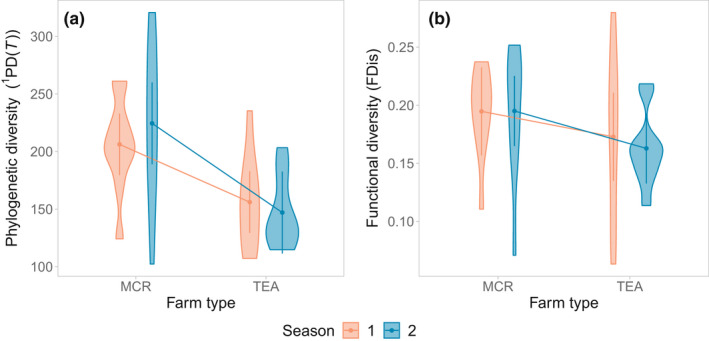
Comparison of (a) avian phylogenetic diversity (^1^PD(*T*)), and (b) avian functional dispersion (FDis) values across two seasons in tea and mixed crop farming systems around Nyungwe NP, Rwanda. Seasons 1 and 2 denote the dry and wet season, respectively. MC: mixed crops. Sample size (*N* = 40 samples) was equal between the two farm types. Each sample comprised 5 adjacent point counts. Statistical differences were tested using a univariate type III repeated‐measure ANOVA. The error bars are based on the model and represent 95% confidence intervals

**FIGURE 5:**
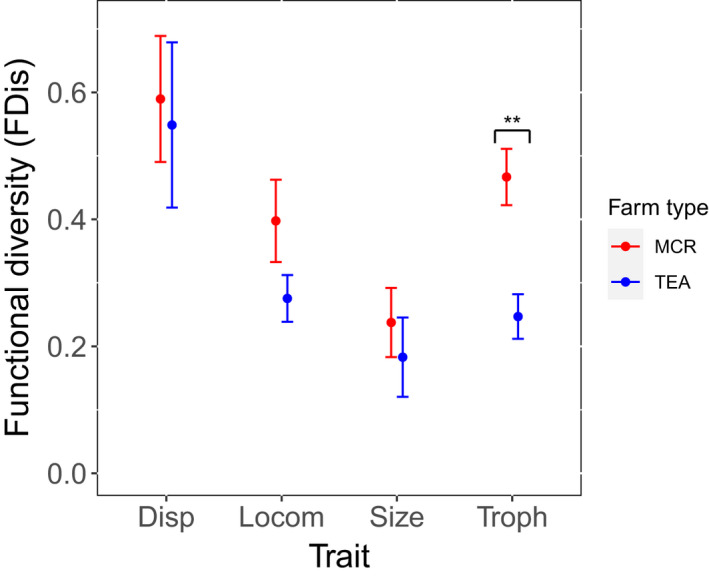
Effects of farming practice on morphological traits of birds found in the Nyungwe NP agricultural matrix, Rwanda (*N* = 20 samples). Functional diversity is calculated as functional dispersion (FDis). MCR: Mixed crops; TEA: TEA plantations. Disp: Dispersal, Locom: Locomotory, Troph: Trophic. Error bars show the standard error of the mean. Statistical differences were tested using a Welch two sample *t*‐test. Asterisks denote significant pairwise differences at *p < .1, **p < .05, and ***p < .01 within the model

Phylogenetic diversity ^1^PD(*T*) differed between farm types (Figure 4). It was significantly higher in mixed crops than in tea farms (*F*_[1, 18]_ = 11.31, *p* = .003). Varying the importance of abundances, the difference between farm types was more pronounced with ^0^PD(*T*), which accounts only for species richness (*F*_[1, 18]_ = 15.56, *p* = .001), and moderate for ^2^PD(*T*), which places more emphasis on the most abundant species (*F*_[1,18]_ = 5.3410, *p* = .0329). Overall FDis did not differ significantly between farm types. There was no effect of season, or of the interaction with farm type, on either PD(*T*) or FDis. A focus on a subset of traits revealed higher FDis values in trophic traits in mixed crops than in tea farms (Figure 5, *t*_[17.11]_ = 3.8854, *p* = .0012). Differences in ses.MNTD values between the farm types were not statistically significant (*t*_[18]_ = 0.841, *p* ≥ .05), however, ses.MPD were close to significance with higher values exhibited by mixed crops than tea farms (*t*_[18]_ = 2.08224, *p* = .0519).

### 3.3 Effects of habitat descriptors on phylogenetic and functional diversity

The most parsimonious model showed crop height and elevation to be important drivers of phylogenetic diversity, while floristic diversity was important for functional diversity. Averaging supporting models within delta AIC_c_ <2 of the model with the lowest AIC_c_ revealed floristic diversity, crop height, elevation, and tree number to be predictors with the most influence (Table 2a). The same predictors were also found to drive functional diversity, with tree number considered the most important factor (Table 2b). Humidity and soil moisture did not have a significant effect on either ^1^PD(*T*) or FDis.

**TABLE 2:** (a and b) Best supported models with ΔAIC_c_ <2 of the model with lowest AIC_c,_ analyzing the influence of farm attributes on (a) ^1^PD(*T*), and (b) FDis of birds sampled in farmlands around Nyungwe NP, Rwanda (*N* = 20 sites). The model average is calculated taking into consideration all initial predictors. The relative importance is obtained by summing the Akaike weights over all models in which the predictor variables appear. Only predictor variables retained in model selection are shown in the Table. 95% confidence intervals (CI) are also given. The small coefficients in b are due to the use of the Gower distance in the computation of FDis scores, which is the recommended distance for mixed variables. Model order (i.e., model no.) is determined by AIC_c_ values

^1^PD(T)							
Model no.	Floristic diversity	Crop height	Elevation	Tree number	AIC_c_	ΔAIC_c_	Weight
1		28.48	−22.43		205.31	0.00	0.089
2	27.67	17.86			205.45	0.14	0.083
3	39.29	0			205.52	0.21	0.080
4	35.01	0		14.02	205.56	0.25	0.079
5		22.68	−23.22	13.5	205.78	0.47	0.070
6	16.63	20.47	−13.89		207.03	1.72	0.038
7	26.94	13.91		10.78	207.14	1.83	0.036
8	25.44	0	−13.39	15.84	207.28	1.97	0.033
**Average**	**20.93**	**13.56**	**−9.07**	**5.83**			
CI‐2.5%	5.54	−0.39	−40.65	−4.13			
CI‐97.5%	55.49	43.99	0.71	31.33			
Importance	0.69	0.62	0.45	0.43			

b. FDis							
Model no.	Tree number	Floristic diversity	Elevation	Crop height	AIC_c_	ΔAIC_c_	Weight
1		0.02			−64.67	0.00	0.26
2	0.02		−0.02		−64.07	0.60	0.19
3	0.01	0.02			−63.82	0.84	0.17
4				0.021	−63.44	1.23	0.14
5	0.02				−63.15	1.52	0.12
6			−0.02		−63.11	1.56	0.12
**Average**	**0.01**	**0.01**	**−0.06**	**0.002**			
CI‐2.5%	−0.01	−0.02	−0.03	−0.01			
CI‐97.5%	0.03	0.03	0.01	0.02			
Importance	0.48	0.43	0.31	0.14			

*Note*: Bold values indicate averages of parameter estimates for each predictor variable.

## 4 DISCUSSION

There was moderate support for the hypothesis that bird species composition differed between tea plantations and mixed crops. Our prediction that avian diversity was higher in mixed crops than in tea plantations was strongly supported for phylogenetic diversity, but not for functional diversity. Analyzing subsets of traits separately revealed less diversity of trophic‐related traits in tea plantations. Floristic diversity, crop height, elevation, and the number of trees were found to be major attributes influencing, to varying degrees, both functional and phylogenetic diversity.

### 4.1 Avian diversity varies with farming practice

Although there was moderate overlap between bird species encountered in tea and mixed crop farms, species in tea farms comprised a high number of rare species in terms of encounter rate, particularly of those species normally associated with forest and forest edge habitats. These birds may have been attracted by the remnant trees and for the case of the afromontane endemic sunbirds, also by Eucalyptus woodlots, whose flowers are increasingly important nectar sources, especially outside forests (Vande weghe & Vande weghe, 2011).

Mixed crops farms harbored twice the number of common bird species (those of ≥10 records) found in tea plantations. Since no significant changes were registered in the community phylogenetic structure as shown by ses.MPD and ses.MNTD values, the loss of phylogenetic diversity in tea farms compared to mixed crops can be explained by the reduction of species. Bird families that were not present in tea plantations, but were associated with mixed crops included Threskiornithidae, Accipitridae, Laniidae, Bucerotidae, and Gruidae. Our findings concur with Frishkoff et al.’s (2014) study in Costa Rica, which found intensive monocultures presented greater probability of species extirpation than more heterogenous agricultural systems.

The maintenance of overall functional diversity in tea plantations relative to mixed crop farms can partly be attributed to the functional redundancy in the latter, which mirrors that often found in natural ecosystems across a range of taxa (Cooke et al., 2019; Edwards et al., 2014; Prescott et al., 2016). For instance, both farm types were dominated by small‐sized insectivorous birds. On the other hand, the absence of functional differences between the farming types can be explained by compensatory dynamics, where disturbance‐tolerant species replace habitat specialists, resulting overall in the maintenance of comparable functional diversity at the community level (Morante‐Filho et al., 2018; Supp & Ernest, 2014). For instance, some of the species only recorded in mixed crop farms had substitutes in tea farms with equivalent functional trait values, among them were: swallows, *Hirundo rustica,* substituted by *Psalidoprocne prisoptera*; pigeons, *Streptopelia semitorquata* by *Turtur tympanistra*; and shrikes, *Lanius mackinnoni* and *L. collaris* replaced by a cuckooshrike, *Coracina caesia* and a tchagra, *Tchagra australis*. These results highlight the value of analyzing different aspects of avian diversity in response to habitat variation. However, the compensatory dynamics reported in this study cannot always be generalized to other human‐modified habitats, as shown by a range of studies that recorded alterations of the functional trait structure of birds that occupy simplified habitats (Bregman et al., 2016; Cannon et al., 2019; Rocha et al., 2019). Such alterations were also found in this study at a finer scale of analysis, as discussed below.

The main difference in functional diversity was found in trophic trait values. Bird communities in tea plantations contained reduced diversity of functional traits, reflecting reduced sources of fruits and grains. This limited the occurrence of frugivores and granivores, including small mammals, and presumably reduced populations of raptors preying on them. Large‐sized birds are often reported to be the first to disappear in agricultural systems, particularly in monocultures, due to greater exposure to hunting (Frishkoff et al., 2014; Thiollay, 2006). The lack of difference in the size traits in our study could be explained by the fact that the hunting of birds in Rwanda has not been a substantial tradition (Vande weghe & Vande weghe, 2011).

### 4.2 Similar habitat attributes drive phylogenetic and functional diversity

We found that avian diversity increased with floristic diversity and height on farmlands as more complex habitat structure and variability permits the coexistence of bird species of diverse lineages and functional traits (Huang et al., 2014; Karr & Roth, 1971; Vollstädt et al., 2017). These findings concur with those of other studies conducted in tropical agricultural systems, which have described how the low niche‐breadth of monocultures restricts understorey birds, affecting diet and foraging stratum traits (Almeida et al., 2016; Azhar et al., 2013; Prescott et al., 2016). The proportion of noncrop vegetation in a farm also positively influences avian diversity and beneficial insects (Carvell et al., 2015; Grass et al., 2016; Lee & Goodale, 2018). In our study, the most diverse farms had the tallest species of noncrop plants, growing on uncultivated strips, resting land, as weeds, or for farm demarcation and medicinal use. The noncrop plants were dominated by the Asteraceae, whose floral morphology attracts large numbers of insects and other invertebrates. Plants grown for farm demarcation also constituted different tree species and shrubs, particularly *Dracaena afromontana*, which provided variation of vertical layers for birds with different strata affinities.

The number of trees was particularly important for avian functional diversity. Some of the fruit‐bearing trees were remnants of the cleared montane rainforest. Such forest remnants are crucial for the maintenance of frugivores and nectivores and thus enhance landscape connectivity and maintain seed dispersal and pollination processes in agricultural systems (Şekercioğlu et al., 2019; Tscharntke et al., 2008). Trees also complement crops by providing upper storey habitats, which are used by canopy dwellers, perching birds, and tree‐nesting birds, hence permitting complementary functional strategies (Kupsch et al., 2019; Şekercioğlu, 2012). The proximity of farms to forests in the tropics has been reported to contribute to the maintenance of avian diversity (Cannon et al., 2019; Raman, 2006), we thus expected to see a negative relationship between phylogenetic and functional diversity and distance from the adjacent 1019 km^2^ Nyungwe forest. The lack of evidence for this effect could be attributed to the extreme loss of forest species in agrosystems and colonization of lineages from a nonforest species pool.

Elevation negatively influences vegetation composition and structural configuration in natural ecosystems (Jankowski et al., 2013; Peters et al., 2016), hence, high negative correlation between elevation and both floristic diversity and crop height should be expected. However, we suggest that human manipulation of agricultural systems, by for instance pruning tea plants at a certain height, or choosing the type and number of crops grown, may have limited this expected effect (Table S1). Furthermore, elevation is factored into the decision‐making processes on the species and number of trees planted on a farm. A study by Mukuralinda et al. (2016) on tree‐based systems in Rwanda found that farmers in high elevation areas preferred planting trees that could supply stakes for climbing beans, for timber, and to help control soil erosion. As a result, trees planted in our study area were dominated by nonindigenous species of *Eucalyptus, Grevillea,* and *Persea*. Nonnative trees in Rwanda are known to support less avian diversity compared to indigenous species (Rurangwa et al., 2021; Vande weghe & Vande weghe, 2011), thus their intentional displacement within high elevation areas could contribute further to elevation effects on the avian diversity.

### 4.3 Landscape management implications

Although the use of electrical fencing might help in deterring animals and in reducing human–wildlife conflicts, the high frequency of forest animals raiding subsistence crops that bear fruits, grains, and tubers in areas around rainforests, encourages the planting of less palatable, commercially valuable monoculture crops, such as tea. Moreover, there are other ongoing incentives to adopt intensive agriculture in Afrotropical landscapes. For example, in Rwanda, since 2008, the “crop intensification program,” has promoted the cultivation of single crops and the application of pesticides and inorganic fertilizers. The expansion of monoculture estates generates detrimental effects on avian diversity and threatens important ecosystem services such as predation, pollination, and seed dispersal, which directly influence farm productivity and cost, as evidenced by research in different parts of the tropics (Gurr et al., 2016; Sinu, 2011). Our research suggests that where tea monoculture is nonetheless a favored agricultural option from an economic perspective, practicing agroforestry guarantees maintenance of good levels of avian diversity, and the meeting of restoration goals pledged by Ministers of African Countries (2016) and their representatives in the “Kigali declaration on forest landscape restoration in Africa.” In the context of conflicting policy desiderata, the impact of agricultural intensification programs and of mitigation schemes to reduce their impact on farmland biodiversity are topics that future studies should investigate for improved agricultural policy guidance.

## AUTHOR CONTRIBUTIONS

**Marie Laure Rurangwa:** Conceptualization (lead); data curation (lead); formal analysis (lead); funding acquisition (lead); methodology (equal); project administration (lead); resources (lead); visualization (lead); writing – original draft (lead); writing – review and editing (equal). **Protais Niyigaba:** Conceptualization (supporting); data curation (lead); formal analysis (supporting); funding acquisition (supporting); investigation (equal); methodology (equal); project administration (equal); resources (equal); validation (equal); writing – review and editing (supporting). **Joseph Andrew Tobias:** Data curation (equal); methodology (equal); resources (supporting); writing – review and editing (equal). **Robert J Whittaker:** Conceptualization (equal); data curation (supporting); formal analysis (equal); funding acquisition (supporting); methodology (equal); project administration (equal); resources (supporting); supervision (lead); validation (lead); visualization (supporting); writing – original draft (equal); writing – review and editing (equal).

## CONFLICT OF INTEREST

The authors declare no potential conflict of interest.

## Supporting information


**Appendix S1** Supporting information

## Data Availability

The data that supports the findings of the functional diversity part of this study are openly available in the Dryad Digital Repository (https://doi.org/10.5061/dryad.41ns1rndd ).The phylogenetic data can be retrieved from www.birdtree.org using the provided list of study species. Restrictions apply to the availability of the rest of the data due to co‐ownership with a third party, so the data are not publicly shared.
