# Supplementary material for: Functional and phylogenetic diversity of an agricultural matrix avifauna: The role of habitat heterogeneity in Afrotropical farmland
Source: Ecol Evol. 2022 Jul 6;12(7):e9024. doi: 10.1002/ece3.9024 (PMC9259849; doi:10.1002/ece3.9024)
Supplement: Supplementary file 1 — Appendix S1 Supporting information [file ECE3-12-e9024-s001.docx]

**Supporting Information**

**Appendix S1. Agricultural intensity**

To determine the intensity and frequency of fertilizers and pesticides used, informal interviews were administered to two local agronomists, and 10 farmers in sites we sampled. We found out that there was generally low application of agro-chemicals in comparison to global standards, in part due to the prohibitive prices of industrial chemicals, and because of the traditional appreciation of organic fertilizers. The wide national donations of domestic animals, especially cows to farmers under the Gira inka munyarwanda program (translates as may each Rwandan have a cow) has also increased access to manure, thus curbing the need for industrial fertilizers.

Permission to conduct the study was sought from local authorities and farmers whose fields we sampled. The research proposal was evaluated and approved by the Central University Research Ethics committee and the Government of Rwanda.

**TABLE S1** Correlation matrix of habitat descriptors recorded in 20 samples constituted of 100 points placed in tea monocultures and mixed crop farmlands around the Nyungwe NP, Rwanda. At each point measurements were taken twice over two seasons except for elevation (Elev) and distance to Nyungwe NP (D.S), which were recorded once.

**Note**: C.HT: Crop height, Temp: Temperature, Hum: Humidity, S.M: Soil moisture, T.H: Tree height, T.N: Tree Number, C.eH: Exponential Shannon-Weiner index of plant species ( floristic diversity), C.rich: Cropland plant species richness, Elev: Elevation, D.S: Distance from sample centroid to Nyungwe NP.

**TABLE S2** List of bird species encountered in sampled farms around the Nyungwe National park, Rwanda, and the type of farm in which they were recorded. MCR denote mixed crops, and both implies the species was recorded in both tea and mixed crops farms. The species nomenclature follows the IOC world bird list, version 8.2. Doi: 10.14344/IOC.ML.8.2.

| **Species name** | **Abbreviations** | **Farm type** |
| --- | --- | --- |
| Anthus trivialis | A.TR | Both |
| Arizelocichla nigriceps | A.NI | MCR |
| Balearica regulorum | B.RE | MCR |
| Bostrychia hagedash | B.HA | MCR |
| Bradypterus cinnamomeus | B.CI | TEA |
| Buteo augur | B.RU | MCR |
| Bycanistes subcylindricus | B.SU | MCR |
| Camaroptera brachyura | C.BR | Both |
| Chalcomitra senegalensis | C.SE | MCR |
| Cinnyris regius | C.RE | TEA |
| Cinnyris stuhlmanni | C.ST | TEA |
| Cinnyris venusta | C.VE | Both |
| Cisticola ayresii | C.AY | Both |
| Cisticola chubbi | C.CH | Both |
| Colius striatus | C.ST | MCR |
| Columba arquatrix | C.AR | Both |
| Coracina caesia | C.CA | TEA |
| Corvus albicollis | C.AL | Both |
| Cossypha caffra | C.CA.1 | TEA |
| Cossypha heuglini | C.HE | MCR |
| Crithagra citrinelloides | C.CI | Both |
| Crithagra striolatus | C.ST.2 | Both |
| Cyanomitra olivacea | C.OL | TEA |
| **Species name** | **Abbreviations** | **Farm type** |
| Dioptrornis fischeri | M.FI | MCR |
| Elminia albiventris | E.AL | MCR |
| Emberiza flaviventris | E.FL | MCR |
| Estrilda kandtii | E.KA | TEA |
| Estrilda nonnula | E.NO | Both |
| Estrilda paludicola | E.PA | Both |
| Estrilda quartinia | C.QU | Both |
| Euplectes capensis | E.CA | MCR |
| Hedydipna collaris | H.CO | TEA |
| Hirundo angolensis | H.AN | Both |
| Hirundo rustica | H.RU | MCR |
| Lagonosticta senegala | L.SE | MCR |
| Lanius collaris | L.CO | MCR |
| Lanius mackinnoni | L.MA | MCR |
| Lonchura cucullata | L.CU | MCR |
| Milvus aegyptius | M.AE | MCR |
| Motacilla aguimp | M.AG | MCR |
| Motacilla capensis | M.CA | MCR |
| Muscicapa adusta | M.AD | MCR |
| Nectarinia kilimensis | N.KI | Both |
| Nectarinia purpureiventris | N.PU | TEA |
| Nigrita canicapillus | N.CA | TEA |
| Onychognathus walleri | O.WA | TEA |
| **Species name** | **Abbreviations** | **Farm type** |
| Parus leucomelas | M.LE | MCR |
| Passer griseus | P.GR | MCR |
| Ploceus baglafecht | P.BA | MCR |
| Ploceus cucullatus | P.CU | MCR |
| Pogoniulus bilineatus | P.BI | MCR |
| Prinia subflava | P.SU | MCR |
| Psalidoprocne pristoptera | P.PR | Both |
| Pycnonotus barbatus | P.BA.1 | Both |
| Saxicola torquatus | S.TO | Both |
| **Species name** | **Abbreviations** | **Farm type** |
| Streptopelia lugens | S.LU | Both |
| Streptopelia semitorquata | S.SE | MCR |
| Tchagra australis | T.AU | TEA |
| Terpsiphone viridis | T.VI | MCR |
| Turdus olivaceus | T.OL | Both |
| Turtur tympanistria | T.TY | TEA |
| Vidua macroura | V.MA | MCR |
| Zosterops senegalensis | Z.SE | Both |

**TABLE S3** ANOSIM pairwise tests for the study samples across seasons. The size of the sample is given in parentheses. Each sample constitutes 5 contiguous points within the same site. 1 and 2 denote the first and second sampling season, respectively. The overall sample statistic R was 0.241. R values close to 1 indicate greater similarity between samples of the same group, and lower similarity between the compared pair. The test was based on 1000 randomizations.

| 1st Group | 2nd Group | P Value | R values |
| --- | --- | --- | --- |
| MCR1 (10) | MCR2 (10) | 0.303 | 0.023 |
| MCR1 (10) | TEA1 (10) | **0.001** | 0.390 |
| MCR1 (10) | TEA2 (10) | **0.003** | 0.265 |
| MCR2 (10) | TEA1 (10) | **0.001** | 0.415 |
| MCR2 (10) | TEA2 (10) | **0.007** | 0.275 |
| TEA1 (10) | TEA2 (10) | 0.123 | 0.088 |

Note: MCR stands for mixed crop farm type


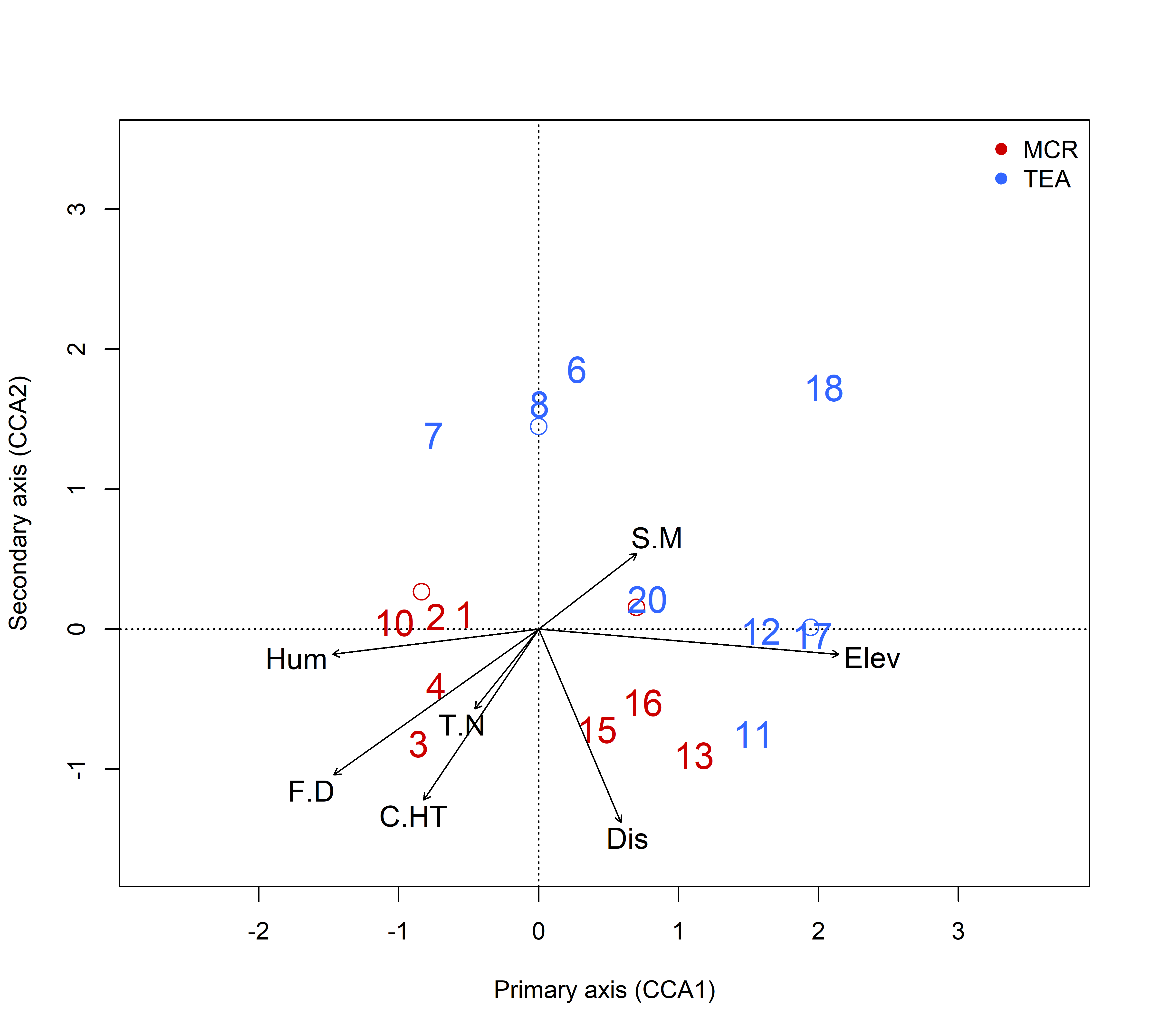


**Figure S1** Constrained Correspondence Analysis plot axis 1 and 2, showing sample sites scores (N = 20), and habitat variables. MCR: Mixed crops, Elev: Elevation, Dis: Distance from forest, C.H Crop height, T.N: Tree number, F.D: floristic diversity, Hum: Humidity, S.M: Soil moisture. The statistical significance of habitat variables were tested by a permutation test, which revealed that elevation was the sole significant environmental variable (P = 0.005) wit
